# Hyperbaric oxygen therapy effects on pulmonary functions: a prospective cohort study

**DOI:** 10.1186/s12890-019-0893-8

**Published:** 2019-08-13

**Authors:** Amir Hadanny, Tal Zubari, Liat Tamir-Adler, Yair Bechor, Gregory Fishlev, Erez Lang, Nir Polak, Jacob Bergan, Mony Friedman, Shai Efrati

**Affiliations:** 1 The Sagol Center for Hyperbaric Medicine and Research, Shamir Medical Center, Zerifin, Israel; 2 Sackler School of Medicine, Tel-Aviv University, Tel-Aviv, Israel; 3 Bar Ilan University, Ramat-Gan, Israel; 4 Faculty of Biomedical Engineering, Technion, Haifa, Israel; 5 Research and Development Unit, Shamir Medical Center, Zerifin, Israel; 6 Sagol School of Neuroscience, Tel-Aviv University, Tel-Aviv, Israel

**Keywords:** Pulmonary function, Oxygen toxicity, Hyperbaric oxygen, HBOT, PFT

## Abstract

**Background:**

Oxygen toxicity is one potential side effect of hyperbaric oxygen therapy (HBOT). Previous small studies showed mild reductions in pulmonary functions reflecting reductions in small airway conductance after repetitive hyperbaric oxygen sessions. However, there are no updated data with well performed pulmonary tests that address the pulmonary effect of the currently used HBOT protocols.

The aim of this study was to evaluate the effect of HBOT on pulmonary functions of patients receiving the currently used HBOT protocol.

**Methods:**

Prospective analysis included patients, 18 years or older, scheduled for 60 daily HBOT sessions between 2016 and 2018. Each session was 90 min of 100% oxygen at 2 ATA with 5 min air breaks every 20 min, 5 days per week. Pulmonary functions, measured at baseline and after HBOT, included forced vital capacity (FVC), forced expiratory volume in 1 sec (FEV1) and peak expiratory flow rate (PEF).

**Results:**

The mean age was 60.36 ± 15.43 and 62.5% (55/88) were males. Most of the patients (83/88, 94.3%) did not have any pulmonary disease prior to inclusion and 30.7% (27/88) had a history of smoking.

Compared to baseline values, at the completion of 60 HBOT sessions, there were no significant changes in FEV1 (0.163), FEV1/FVC ratio (0.953) and FEF25–75% (0.423). There was a statistically significant increase though not clinically relevant increase in FVC (0.1 ± 0.38 l) and PEF (0.5 ± 1.4 l) with a 0.014 and 0.001 respectively.

**Conclusion:**

Regarding pulmonary functions, repeated hyperbaric oxygen exposure based on the currently used HBOT protocol is safe. Surprisingly, there was a modest non clinically significant though statistically significant improvement in PEF and FVC in the current cohort of patients who were without chronic lung diseases.

**Trial registration:**

Clinicaltrials.gov, trial ID: NCT03754985, (Nov 2018) Retrospectively registered.

## Take home message

- Repeated hyperbaric oxygen sessions of 90 min at 2 ATA, with 5 min air breaks every 20 min, are safe and have no negative impact on pulmonary functions.
- The currently used HBOT protocol induces a modest improvement in PEF and FVC in patients without chronic lung diseases.

## Background

Oxygen is a vital but potent biochemical and as such, it can be toxic depending on dose and exposure duration [1]. Although oxygen-induced damage has been reported in most tissues, the lungs are mostly affected since the oxygen partial pressure (PO_2_) is the highest in the alveoli. Pulmonary oxygen toxicity manifests as two overlapping phases as seen in pathology. The acute exudative phase includes interstitial and alveolar edema, hemorrhage, inflammation and fibrinous exudate along with injury to the endothelial cells and type I alveolar cells. The subacute proliferative phase is defined by interstitial fibrosis, fibroblastic proliferation, and hyperplasia of type II alveolar cells [2]. The first sign of pulmonary toxicity is tracheobronchial irritation, which is clinically expressed as substernal or pleuritic pain [1] followed by a decrease in pulmonary function in both the acute and subacute proliferative phase [3].

Clarke et al.’s novel studies show that the incidence of pulmonary oxygen toxicity increases both with the inspiratory partial pressure and the time of exposure in a continuous single exposure [4, 5]. In normal humans, toxicity can be expected after about 10 h of 100% oxygen at 1ATA, after 8–14 h at 1.5 ATA and 3–6 h at 2 ATA of continuous exposure with symptoms subsiding after 4 hours [6].

Hyperbaric oxygen therapy (HBOT), which utilizes both high pressure and high concentrations of oxygen, in multiple daily sessions per patient, has the potential to induce pulmonary oxygen toxicity. Two previous studies evaluated the effects of repetitive HBOT on pulmonary function. Pott et al. [7] studied 14 patients who underwent 30 daily sessions of 90 min exposure to 2.4 ATA pure oxygen in a monoplace chamber. Patients were exposed to hyperoxia without any air breaks during the sessions. There were no significant changes in forced vital capacity (FVC) or diffusing capacity. Furthermore, most of the patients in this study were heavy smokers with impaired diffusing capacity at baseline. Thorsen et al. [8] included 20 patients who underwent 21 repetitive daily HBOT sessions. The protocol included 90 min of 2.4 ATA 100% oxygen in 3 cycles of 30 min each, separated by two 5 min breaks where patients breathed air in-between (“air breaks”). There were no significant changes in FVC, forced expiratory flow at 25% of pulmonary volume (FEF25%) or peak expiratory flow rate (PEF). However, there were significant reductions in forced expiratory volume in 1 second (FEV1). No significant change was noticed in diffusing capacity.

In recent years, HBOT is used for a growing number of patients for new indications, using protocols that are based on lower oxygen pressure (2 ATA or less), but for a prolonged period with more daily sessions (40–60 sessions). The new emerging indications are mostly neurological, including idiopathic sudden sensorineural hearing loss [9], post-stroke and post-traumatic brain injury [10–12], post-radiation injury, as well as chronic pain such as fibromyalgia syndrome [13]. In these conditions, the protocols are based on 40–60 HBOT daily sessions. The safety of these longer protocols regarding pulmonary oxygen toxicity hasn’t been well evaluated yet.

The aim of the current study was to assess pulmonary oxygen toxicity as measured by pulmonary functions associated with 60 daily sessions of HBOT.

## Methods

The study was performed as a prospective cohort study conducted at the Sagol Center for Hyperbaric Medicine and Research at Assaf Harofeh Medical Center between February 2016 and June 2018. The protocol was approved by our institution’s institutional review board (IRB) (0024–16-ASF). All participants signed written informed consent prior to their inclusion.

### Participants

The study included participants 18 years or older, scheduled for 60 HBOT sessions for any indication. Active smokers were excluded but patients who quit smoking more than 6 months prior to inclusion were allowed in the study.

Exclusion criteria included active smoking, severe known pulmonary disease, chest pathology incompatible with HBOT, inner ear disease, claustrophobia, other neurological conditions, pregnancy, previous HBOT within 6 months prior to inclusion and the inability to sign informed consent. Patients who did not complete 60 sessions due to non-pulmonary reasons were excluded as well.

Data collected from the patient’s medical files included age, gender, chronic medical conditions, medications, previous smoking and indication for HBOT therapy.

### Protocol

After signing an informed consent form, the participants underwent a pulmonary function baseline evaluation. Participants were treated in a multiplace chamber (HAUX-Life-Support GmbH) for 60 daily sessions, 5 days a week. Each session consisted of 90 min of exposure to 100% oxygen at 2 ATA with 5 min air breaks every 20 min. Participants repeated their pulmonary function evaluation after the last HBOT session. Measurements were taken in the morning before entering the hyperbaric chamber of the first and last session (22–23 h post the previous session).

### Pulmonary function

Measurements of pulmonary functions were performed using the MiniSpir testing apparatus (MIR- Medical International Research, USA). The equipment was calibrated using a 3-l syringe before performing measurements according to the manufacturer’s instructions. Measurements were performed by a trained technician. The forced expiratory maneuvers were performed as recommended by the guidelines [14].

The forced vital capacity (FVC), forced expiratory volume in 1 sec (FEV1) and peak expiratory flow rate (PEF) were taken as the highest readings obtained from at least three satisfactory forced expiratory maneuvers. Mean forced mid-expiratory flow rate (FEF25–75%) and forced expiratory flow rates at 25, 50 and 75% of FVC expired (FEF25%, FEF50% and FEF75%) were taken as the best values from flow–volume loops not differing by > 5% from the highest FVC. Pulmonary function was analyzed using WinSpiroPRO with Knudson reference values [15].

### Sample size

The expected mean changes in FVC in repeated spirometry of adults are 72 ± 76 ml [16]. Using alpha of 0.05, the mean change in FVC in the current study with sample size of 88 patients gives a power of 93.3% .

### Statistical analysis

Continuous data were expressed as means ±standard deviations. The normal distribution for all variables was tested using the Kolmogorov-Smirnov test. Dependent t-tests were performed to compare changes within groups. Possible covariates (age, gender, biometric data, chronic medical conditions, medications) were analyzed using a general linear model. Categorical data were expressed in numbers and percentages and compared by chi-square tests. Univariate analysis was performed using Chi-Square/Fisher’s exact test (where appropriate) or to identify significant variables (*P* < 0.05). The alpha level was set to 0.05. Data were statistically analyzed using SPSS software (version 22.0).

## Results

**Fig. 1:**
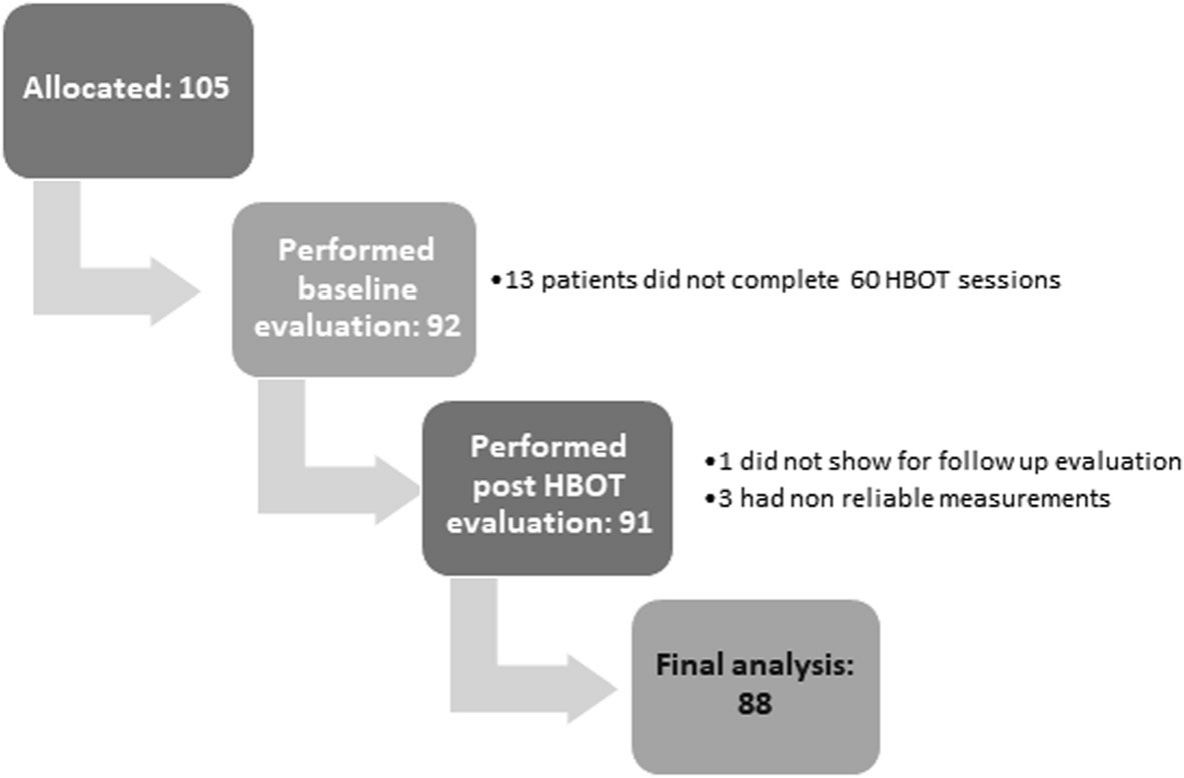
Patients flowchart: out of 105 patients, 88 patients were included in the final analysis

Between February 2016 and June 2018, 105 patients signed informed consents and performed baseline evaluations. Thirteen patients did not complete 60 HBOT sessions and were excluded from anaylsis. Three patients in whom one of the tests was performed without pre-test system calibration and one patient who did not complete the post HBOT evaluation were also excluded from final analysis. Accordingly, 88 patients were included in the final analysis (Fig. 1).

The mean age was 60.36 ± 15.43 and 62.5% (55/88) were males. Most of the patients (83/88, 94.3%) did not have any pulmonary disease prior to inclusion and 30.7% (27/88) had a history of smoking. Most patients were treated for neurological indications (71.6%, 63/88). There were significant differences in biometric measurements, chronic medical conditions, prescribed drugs and baseline pulmonary functions. See Table 1 for basic characteristics.

**Table 1:** Patients baseline characteristics

	Total	Males	Females	Significance
N	88 (100%)	55 (62.5%)	33 (37.5%)	
Age (years)	60.36 ± 15.43	±11.26 65.13	52.41 ± 18.15	**0.0001**
Height (cms)	170.72 ± 9.72	175.63 ± 6.54	162.54 ± 8.62	**< 0.0001**
Weight (kgs)	75.20 ± 14.69	81.94 ± 13.16	63.96 ± 9.29	**< 0.0001**
BMI	25.67 ± 3.79	26.54 ± 3.85	24.25 ± 3.28	**0.005**
Chronic medical conditions				
Diabetes mellitus	17 (19.3%)	15 (27.3%)	2 (6.1%)	**0.015**
Hypertension	26 (29.5%)	22 (40%)	4 (12.1%)	**0.006**
Hypercholesterolemia	26 (29.5%)	21 (38.2%)	5 (15.2%)	**0.022**
Ischemic heart disease	13 (14.8%)	13 (23.6%)	0	**0.001**
Pulmonary disease	5 (5.7%)	5 (9.1%)	0	0.15
History of smoking	27 (30.7%)	21 (38.2%)	6 (18.8%)	0.059
Pack years (in smokers)	18.33 ± 13.95	19.31 ± 15.15	14.91 ± 8.72	0.51
Indication				0.707
Neurological	(65.9%) 58	36 (65.4%)	22 (66.6%)	
Wounds/Radiation	6 (8.4%)	3 (5.5%)	3 (9.1%)	
Other	24 (27.3%)	16 (29.0%)	8 (24.2%)	
Medications				
Anti-aggregation	27 (30.7%)	21 (38.2%)	6 (18.2%)	**0.049**
ACE-Inhibitors	17 (19.3%)	15 (27.3%)	2 (6.1%)	**0.023**
Statins	34 (38.6%)	27 (49.1%)	7 (21.2%)	**0.009**
Proton pump inhibitors	12 (13.6%)	9 (16.4%)	3 (9.1%)	0.336
Baseline Pulmonary functions				
FEV1	2.83 ± 0.73	2.97 ± 0.77	2.60 ± 0.61	**0.022**
FVC	3.55 ± 0.97	3.79 ± 0.96	3.15 ± 0.87	**0.002**
FEV1/FVC	80.10 ± 9.64	78.44 ± 10.1	82.87 ± 8.26	**0.036**
PEF	5.74 ± 1.88	6.87 ± 2.0	5.11 ± 1.57	**< 0.001**

everything under 0.05 are in bold

At baseline, FVC was 3.55 ± 0.97 l or 96.15 ± 19.33% of the predicted values and FEV1 was 2.83 ± 0.73 l or 95.54 ± 20.36%. The FEV1/FVC ratio was 80.10 ± 9.64 or 101.56 ± 13.56 of the predicted values (Table 2). After HBOT, there were no significant changes in FEV1, the FEV1/FVC ratio and FEF25–75 (Table 2, *p* > 0.05). There was a small statistically significant increase of 0.04 ± 0.28 l in FVC, and 0.48 ± 1.39 l/min in PEF (*p* < 0.05) (Table 2).

**Table 2:** Pulmonary function pre and post HBOT

	Baseline	Post HBOT	Mean Change	Significance
FEV1 (l)	2.83 ± 0.73	2.88 ± 0.75	0.04 ± 0.28	0.163
Predicted (%)	95.54 ± 20.36	97.28 ± 20.17	1.74 ± 9.90	0.102
FVC (l)	3.55 ± 0.97	3.65 ± 1.04	0.1 ± 0.38	**0.014**
Predicted (%)	96.15 ± 19.33	98.55 ± 20.17	2.40 ± 9.95	**0.026**
FEV1/FVC	80.10 ± 9.64	79.51 ± 9.23	(−0.60) ± 7.21	0.435
Predicted (%)	101.56 ± 13.56	100.97 ± 13.4	(−0.59) ± 9.01	0.536
FEF2575 (l)	2.94 ± 1.02	2.89 ± 1.00	(−0.04 ± 0.57)	0.423
Predicted (%)	93.86 ± 31.10	92.70 ± 33.37	(−1.15 ± 20.37)	0.597
PEF (l/min)	5.74 ± 1.88	6.23 ± 2.03	0.48 ± 1.39	**0.002**
Predicted (%)	78.71 ± 24.41	84.28 ± 24.20	5.56 ± 18.88	**0.007**

everything under 0.05 are in bold

Age, gender, weight, height, BMI, chronic medical conditions, medications and HBOT indication were not associated with post HBOT FVC, FEV1, FEV1/FVC, FEF25–75 and PEF (*p* > 0.05). Pre HBOT-measurements were the only significant predictor for post HBOT values.

Clinically, none of the patients complained of any cough, irritation, dyspnea or chest pain during and post HBOT.

## Discussion

In this currently largest prospective cohort study of 88 participants, repeated exposures of 60 daily sessions in 2 ATA 100% oxygen had no significant effect on FEV1, FEV1/FVC and FEF25–75, and a small though statistically significant improvement in both FVC and PEF. Thus, even though current HBOT protocols include a higher number of daily sessions, they did not result in pulmonary toxicity.

The mean increase in FVC of 2.8% from baseline after 60 hyperbaric oxygen daily sessions, is relatively small and clinically insignificant. One can claim that the increase can be attributed to training or learning to perform the forced expiratory even though 3 months have elapsed between baseline and post HBOT evaluations. However, if it was attributed to maneuver training, it would have also resulted in an increase in FEV1, which was not seen.. The increase in PEF, which is an effort dependent index, even though found in 3-month interval, may theoretically be related to the learning effect of the patient repeating the test. Enright et al. showed a change of 5.7% inf PEF in repeated tests [16].

Two previous studies had conflicting results regarding the pulmonary toxicity effects of repetitive HBOT sessions. In the Pott et al. study [7] in which 18 patients had 30 daily sessions (Table 3) in a monoplace chamber for 90 min at 2.4 ATA oxygen without air breaks, no significant changes in FVC or diffusing capacity were noticed. However, the Pott et al. study had a considerably small sample, where only 14 patients completed more than 20 sessions. The standard deviations of pulmonary functions were considerably large (over 20%), thus small changes could have been missed. The second study done by Thorsen et al. [8], included 20 patients treated in 21 HBOT daily sessions for 90 min of 2.4 ATA with 30:5 air breaks (Table 3), no significant changes in FVC, FEF25%, PEF or diffusing capacity were noticed. However, there were significant reductions in FEV1 and FEF50–75%.

**Table 3:** Units of pulmonary toxicity dose (UPTD) in HBOT studies

	UPTD per session	Number of sessions	Total UPTD
Hadanny, Efrati et al.	224	60	13,489
Pott et al.	273	30	8213
Thorsen et al.	273	21	5749

Compared to those studies (Table 3), the current study has two main strengths. First, this is the largest prospective sample size (*N* = 88). Post-hoc sample power analysis found the current study has a power of 93.3%. Second, the currently used protocol is significantly longer (60 sessions) and suitable for the updated clinical use of HBOT.

One of the theories regarding the oxygen toxicity mechanism relies on the generation free radicals, which are the byproducts of the respiratory chain for adenosine triphosphate (ATP) production by the mitochondria. Harabin et al. [17] showed that intermittent delivery of HBOT (relatively long periods of hyperoxia interrupted by short periods of low oxygen pressure or normoxia) reduces pulmonary toxicity in animal models. The mechanism of tolerance is mediated by inducing the lung enzyme superoxide dismutase (SOD), which functions as a free radicals scavenger [17]. Therefore, even though HBOT may increase free radical production, intermittent exposure actually induces SOD and other scavengers that reduce the net free radical concentrations and their potential toxic effects.

Arieli et al. [18] calculated a power equation for pulmonary oxygen toxicity, as measured by vital capacity reduction:

$$ \%\Delta \mathrm{VC}\kern0.5em =\kern0.5em 0.0082\kern0.5em \times \kern0.5em {\mathrm{t}}^2\kern0.5em \times \kern0.5em {\left({\mathrm{PO}}_2\right)}^{4.57} $$

Using our HBOT protocol variables of 2 ATA for 90 min:

ΔVC = 0.4382% after a single exposure.

Next, the recovery from pulmonary oxygen toxicity of the hyperbaric exposure was calculated using [18]:

$$ \Delta \mathrm{VCtr}\kern0.5em \%\kern0.5em =\kern0.5em \Delta \mathrm{VCe}\%\kern0.5em \times \kern0.5em {\mathrm{e}}^{-\kern0.5em \left(-\kern0.5em 0.42\kern0.5em +\kern0.5em 3.83927\kern0.5em \times \kern0.5em {\left({\mathrm{PO}}_2\right)}_{\mathrm{e}\mathrm{x}}\right)\times \mathrm{tr}} $$

where tr is the recovery time in hours. ΔVCtr is the value after the recovery time, ΔVCe is the value following the previous hyperbaric oxygen exposure, and PO_2_ex is the previous exposure to hyperbaric oxygen in ATA. The rate of recovery depends on the PO_2_ which caused the insult.

Using a recovery time of 22.5 h after one hyperbaric session, ΔVCtr is zeroed.

Thus, considering our protocol of 60 daily sessions, separated by ~ 24 h, ΔVC results in a complete recovery between sessions. Moreover, using daily sessions at 90 min of 2 ATA, no pulmonary toxicity is expected regardless of the number of sessions. In addition to vital capacity recover, as discussed previously, the levels of scavengers enzymes might still be higher than normal.

The current study has several limitations, First, no control group was used. However, a previous study on 46 healthy males that evaluated the test re-test variability over a period of 3 months, showed that there were no significant changes in FEV1, FVC, FEV1/FVC, PEF and FEF25–75 [19]. Second, lung capacity and diffusion tests were not performed in the study. In the previous studies mentioned above [7, 8], there were no changes in these functions. Third, patients suffering from significant chronic lung diseases were not included in the study. Fourth, spirometry is not sensitive enough to detect small (alveolar) changes. Fifth, the baseline weight and height of the patients were used for all measurements.

## Conclusions

With regards to pulmonary functions, the currently used HBOT protocol that includes 60 daily sessions of 90 min exposure to 100% oxygen at 2 ATA, with 5 min air breaks every 20 min, has no negative effects on pulmonary functions. Surprisingly, there was a modest non clinically significant thought statistically significant improvement in PEF and FVC in the current cohort of patients without chronic lung disease. Further studies are needed for patients with lung diseases.

## Data Availability

The datasets used and/or analysed during the current study are available from the corresponding author on reasonable request.

## Abbreviations

ATA: Absolute atmospheres
FEF25%: Forced expiratory flow at 25% of pulmonary volume
FEF25–75%: Forced mid-expiratory flow rate
FEV1: Forced expiratory volume in one second
FVC: Forced vital capacity
HBOT: Hyperbaric oxygen therapy
PEF: Peak expiratory flow rate
